# Effects of Square Stepping Exercise on cognitive, physical, psychological, and group functioning in sedentary older adults: A center-based hybrid trial

**DOI:** 10.1186/s12877-024-04904-7

**Published:** 2024-04-25

**Authors:** Masato Kawabata, Su Ren Gan, Annabel Chen Shen-Hsing

**Affiliations:** 1 Rikkyo University, College of Sport and Wellness, 1-2-26 Kitano, Niiza, Saitama 352-8558 Japan; 2 The University of Queensland, School of Human Movement and Nutrition Sciences, St Lucia QLD, 4072 Australia; 3 Nanyang Technological University, National Institute of Education, 1 Nanyang Walk, Singapore, 637616 Singapore; 4 Nanyang Technological University, School of Social Sciences, 48 Nanyang Avenue, Singapore, 639818 Singapore; 5 Nanyang Technological University, Centre for Research and Development in Learning (CRADLE), 61 Nanyang Drive, Singapore, 637335 Singapore; 6 Nanyang Technological University, Lee Kong Chian School of Medicine, 11 Mandalay Road, Singapore, 308232 Singapore

**Keywords:** COVID-19, Executive function, Group cohesion, Integrated exercise, Hybrid intervention

## Abstract

**Background:**

The Square Stepping Exercise (SSE) is an exercise training program that integrates physical exercise and cognitive elements and can be conducted in a group setting. The potential of SSE in delaying cognitive decline in older adults is promising. However, the coronavirus pandemic has made it more difficult for older adults worldwide to exercise together in person. To address this issue, this study conducted a wholistic evaluation of the effects of a center-based hybrid SSE trial on cognitive, physical, psychological, and group functioning in sedentary older adults.

**Methods:**

A total of 93 older adults (19 men, 74 women) participated in the study. Fifty-eight participants (9 men, 49 women) completed center-based hybrid SSE sessions over 12 weeks under coronavirus pandemic circumstances, whereas other 35 participants in the control group maintained their current level of daily activities. Cognitive functions focused on executive functions assessed by the Stroop Color-Word Test (inhibition) and the Trail Marking Test (TMT) (set-shifting). Psychological and group functioning were assessed by the Subjective Vitality Scale and the Physical Activity Group Environment Questionnaire. Physical function was evaluated by measuring gait speeds. A repeated ANOVA was conducted on the measured variables separately for the intervention and control groups to focus on the change of participant’s performance over data collection points.

**Results:**

Outcomes of the Stroop Color-Word Test and the TMT revealed that the hybrid SSE was highly effective in improving executive function. Stroop performance (correct trials) was significantly improved in the incongruent condition, as well as both TMT-A and -B over the intervention period in the intervention group. The hybrid SSE was also beneficial to improve physical (gait speed at usual pace and at the maximum pace) as well as psychological functioning (subjective vitality). Furthermore, SSE participants reported increased engagement with the SSE task, social communication, and increased bonding and closeness with their group members through the hybrid SSE.

**Conclusions:**

In this study, hybrid SSE was found to be effective in enhancing cognitive, physical, psychological, and group functioning in sedentary older adults. The findings of this study are crucial to provide older adults with a safer and efficient option to exercise.

The world’s population is aging much faster than in the past, and the population of people aged 60 years and above will be 2.1 billion across the world by 2050 [1]. To make the additional years of life positive for older people and for society, it is crucial to maintain physical and mental capacity of older adults. Karr et al. [2] have proposed that integrating both physical exercise and cognitive training in an intervention program may be more advantageous to maximize the benefits of training programs.

A training program that consists of both physical exercise and cognitive elements is the Square Stepping Exercise (SSE) [3]. SSE is a type of stepping exercise training that can be easily conducted in a group environment. The SSE consists of multi-direction movements, involving forward, backward, lateral and oblique step patterns. They are classified into 3 levels (elementary, intermediate, and advanced) based on the difficulty of the stepping patterns [3, 4, 15]. It was developed for older adults to do exercise indoor by overcoming difficulties faced when walking outdoors. Furthermore, SSE has the potential to support social interactions when it is done in a group environment [3].

Previous research showed that SSE was efficient to reduce fall risks in healthy community dwelling older adults [3–5] and to increase gait abilities and balancing of older adults with Parkinson’s disease [6]. Meta-analytic reviews [7, 8] also supported the effectiveness of SSE on fall prevention by enhancing balance [7, 8]. In addition to the physical benefits, the SSE was found to provide older adults with psychological benefits (e.g., improving depressive signs [9]) and cognitive benefits [10–12]. Teixeria et al. [10] found that a 14-week SSE program was effective in enhancing attention, global cognition, and mental flexibility [10], whereas other researchers found cognitive gains in executive function and memory [11, 12]).

The potential of SSE in delaying cognitive decline in older adults is promising [9–11]. However, the coronavirus pandemic has made it more difficult for older adults worldwide to exercise together in person. Individuals aged 60 years and over are more vulnerable to the coronavirus and at higher risk of developing serious conditions [13]. Considering the coronavirus pandemic, it was important to explore the option of the online SSE method for older adults. Kawabata et al. [15] invented protocols to conduct a home-based SSE trial online to increase the safety of participants. They reported that the online SSE was effective in improving executive function and group cohesion in sedentary young adults. Subsequently, Gan et al. [16] conducted a home-based online SSE with sedentary older adults and investigated its short-term effects on cognitive and group functioning by comparing with those of in-person SSE. They reported that the home-based online SSE was successful in enhancing executive function and group cohesion in sedentary older adults. However, the sample size of Gan et al’s study was very limited (online SSE: *n* = 7; in-person SSE: *n* = 17).

Given that the coronavirus pandemic has been stable but we need to live with the coronavirus, it would be useful to explore another option of the SSE approach for older adults. Therefore, the present study examined the effects of a hybrid SSE trial on cognitive, physical, psychological, and group functioning in sedentary older adults, hypothesizing significant positive outcomes in the intervention group compared to controls.

## Methods

### Participants and procedures

A total of 93 older adults (19 men, 74 women; *M*_*age*_ = 68.3 years, *SD* = 5.7) participated in the present study. The participants were healthy sedentary older adults without physical injuries or medical illness, according to the recruitment criteria indicated below. Fifty-eight participants (9 men, 49 women) in the intervention group were members of senior activity centers, who had never participated in SSE before. They completed hybrid SSE sessions at their centers. Thirty-five participants (10 men, 25 women) in the control group were recruited from online social media platforms. They were instructed to maintain their current level of physical activity during the study period without starting new activities. Participants’ characteristics are presented for each group in Table 1. Participants were recruited for intervention and control groups separately with the following inclusion criteria: (a) no psychiatric or neurological illnesses, (b) aged between 55 and 80 years old; and (c) exercise less than three times per week and not more than 30 min each time [16]. Consequently, participants were not randomly allocated to the groups in this study.

**Table 1:** Characteristics of Participants

	Total (*N* = 93)	Intervention (*n* = 58)	Control (*n* = 35)	p-value
		Men (*n* = 9) Women (*n* = 49)	Men (*n* = 10) Women (*n* = 25)	
Age (years)	68.27 (± 5.70)	70.48 (± 4.60)	64.60 (± 5.50)	*p* <.001
Education Level				*p* <.001
Nil	1 (1.1%)	1 (1.7%)	0 (0.0%)	
Primary	21 (22.6%)	18 (31.0%)	3 (8.6%)	
Secondary	49 (52.7%)	37 (63.8%)	12 (34.3%)	
Diploma	10 (10.8%)	2 (3.4%)	8 (22.9%)	
Bachelors	8 (8.6%)	0 (0.0%)	8 (22.9%)	
Masters	3 (3.2%)	0 (0.0%)	3 (0.0%)	
PhD	1 (1.1%)	0 (0.0%)	1 (2.9%)	
Total Estimated Years of Education	10.22 (± 3.53)	8.69 (± 2.32)	12.74 (± 3.76)	*p* <.001

*Note.* Data of age and total estimated years of education are *Mean* (*SD*) and data of education level are frequencies (%). Total years of education were estimated as follows: Primary (6 years), Secondary (10 years), Diploma (13 years), Bachelors (16 years), Masters (18 years), and PhD (22 years)

Ethical approval was obtained from an Institutional Review Board prior to data collection. Participation in the study was voluntary and informed consent was obtained from every participant. This study was conducted based on the approved guidelines and procedures over a 2-year period (from early 2022 to April 2023). At that time, the coronavirus situation was stable in Singapore and safe management measures were further eased by removing group size limits [14].

### Exercise Protocol

#### Hybrid Square Stepping Exercise

The exercise sessions were conducted in hybrid mode through Zoom, an online video meeting software, twice a week over 12 weeks. Participants met in person at their respective senior activity center and participated in the SSE together in a group setting, but the exercise instruction was provided by an instructor online. As the participants were older adults, precautions against coronavirus were taken in this study. For example, the exercise sessions were limited to the members of each senior activity center. Participants also had to report to the staff whether they had symptoms. If they were feeling unwell, they were advised to stay home. All SSE sessions were scheduled in the morning.

SSE was performed on a thin felt mat that is 2.5 m in length (100 cm × 250 cm) and partitioned into 40 squares (25 cm each). At the start of each round, a certified trainer demonstrated a stepping pattern online to participants by following Kawabata et al.’s study [15]. Participants were asked to remember the pattern shown on the screen. Then, they stepped from one end of the mat to the other by following the stepping pattern. They took turns to execute the pattern presented in each round. Once finished, participants returned to the initial position by walking outside the mat. Positive social interactions were promoted during the SSE session through social communications with verbal encouragements and engagement of ‘air’ high-fives between participants on completion of trials.

### Measures

Participants in both intervention and control groups were asked to complete the following measures (except for group cohesion) before, in the middle, and after the 12-week study period. The measure of group cohesion was administered only to the participants in the intervention group before and after the intervention.

#### Executive function

The Stroop Color-Word Test and the Trail Making Test (TMT) was administered using Inquisit 5 [17] to measure speed of cognitive processing, cognitive flexibility and inhibition [18]. In the Stroop Color-Word Test, cognitive interference (inhibition) was simulated by asking participants to name the color of the word and not read the word itself. The test consists of three different conditions: (i) congruent trials; (ii) incongruent trials; and (iii) control trials [19]. The Stroop congruent and incongruent conditions are the indices of basic information processing and executive function, respectively [19]. The TMT comprised two parts (A and B). Part A (TMT-A) is associated with visuomotor processing speed and Part B (TMT-B) is considered to reflect working memory and task-switching ability [17, 20]. The difference in time taken to complete TMT-B and the time to complete TMT-A (i.e., B– A) was calculated to measure executive function [19, 20].

#### Group cohesion

The Physical Activity Group Environment Questionnaire (PAGEQ) [22] was employed to assess participant’s experience of cohesion in their physical activity groups. The PAGEQ is an instrument with 21 items, consisting of the four subscales: Individual Attractions to the Group-Task (ATG-T: personal participation in the group task), Individual Attractions to the Group-Social (ATG-S: personal approval and social communication with the group), Group Integration-Task (GI-T: the bonding and closeness that exists within the group as a whole around its joint task), and Group Integration-Social (GI-S: the bonding and closeness that exist within the group as a whole around social matters). Participants were requested to show the degree to which they agreed with the statement of each item by using a 9-point Likert-type scale, which ranged from 1 (*very strongly disagree*) to 9 (*very strongly agree*).

#### Subjective vitality

The Subjective Vitality Scale (SVS) [23] is a self-report instrument that is designed to evaluate state and trait feelings of energy and vitality. When people are vital, they are more active and productive, coping better with stress and challenges, and have greater mental health and wellness [24]. The SVS has been extensively used in the research on health, motivation, and well-being [25]. The five-item trait-version of the SVS was employed in the current study as it was found to be a sensitive measure in Kawabata et al. [25]. Participants were requested to indicate the degree to which the statement of each item was true for them “in general in their life” based on a 7-point Likert-type scale, which ranged from 1 (*not at all true*) to 7 (*very true*).

#### Physical tests

A walk and a single-leg balance test were used to measure participant’s physical functioning abilities. For the gait test, participants had to walk along a straight 11-metre walkway at their (1) usual pace once, and (2) maximum pace twice (the faster measurement was taken) [26]. In the balance test, participants were asked to balance on either their left or right leg with their eyes open and hands crossed over their chest; the time they were able to maintain balance in this position was measured [27]. The time begins when they raise their foot and ends when participants attempt to regain their balance by (1) using their arms, (2) placing their raised foot down, or (3) shifting the weight bearing foot. The test also ended when they were able to maintain their balance for the maximum duration of 30s.

### Data Analysis

In preliminary analysis, participants in the control group were found to be significantly younger and more educated than the intervention group (reported in the preliminary results in the Results section later). Therefore, a repeated ANOVA was conducted on the measured variables separately for the intervention and control groups to focus on the change of participant’s performance over data collection time points. A repeated MANOVA was conducted on the PAGEQ subscale scores, which were only applicable to the intervention group. When pairwise comparison was performed as part of the *post hoc* test, Bonferroni-adjusted *p*-values (0.017 [0.05/3]) were used to identify statistical difference between time points (baseline, middle, post) by controlling the overall Type I error rate.

## Results

### Preliminary analysis

Descriptive statistics of participant’s age and frequencies of participant’s education level are presented in Table 1. Participants in the control group (*M* = 68.3, *SD* = 5.71) were significantly younger than those in the intervention group (*M* = 70.5, *SD* = 4.61; *t*[91] = 5.55, *p* <.001, *d* = 1.16). Furthermore, participants in the control group were more educated, compared to those in the intervention group (χ^2^ = 36.62, *p* <.001; total estimated years of education: *t*[91] = 6.44, *p* <.001, *d* = 1.38). According to these results, statistical comparison between groups would be questionable. Instead, focusing on the change of participant’s performance over time in each group was considered more appropriate and meaningful.

### Executive function

#### Stroop Performance in the incongruent condition

**Fig. 1:**
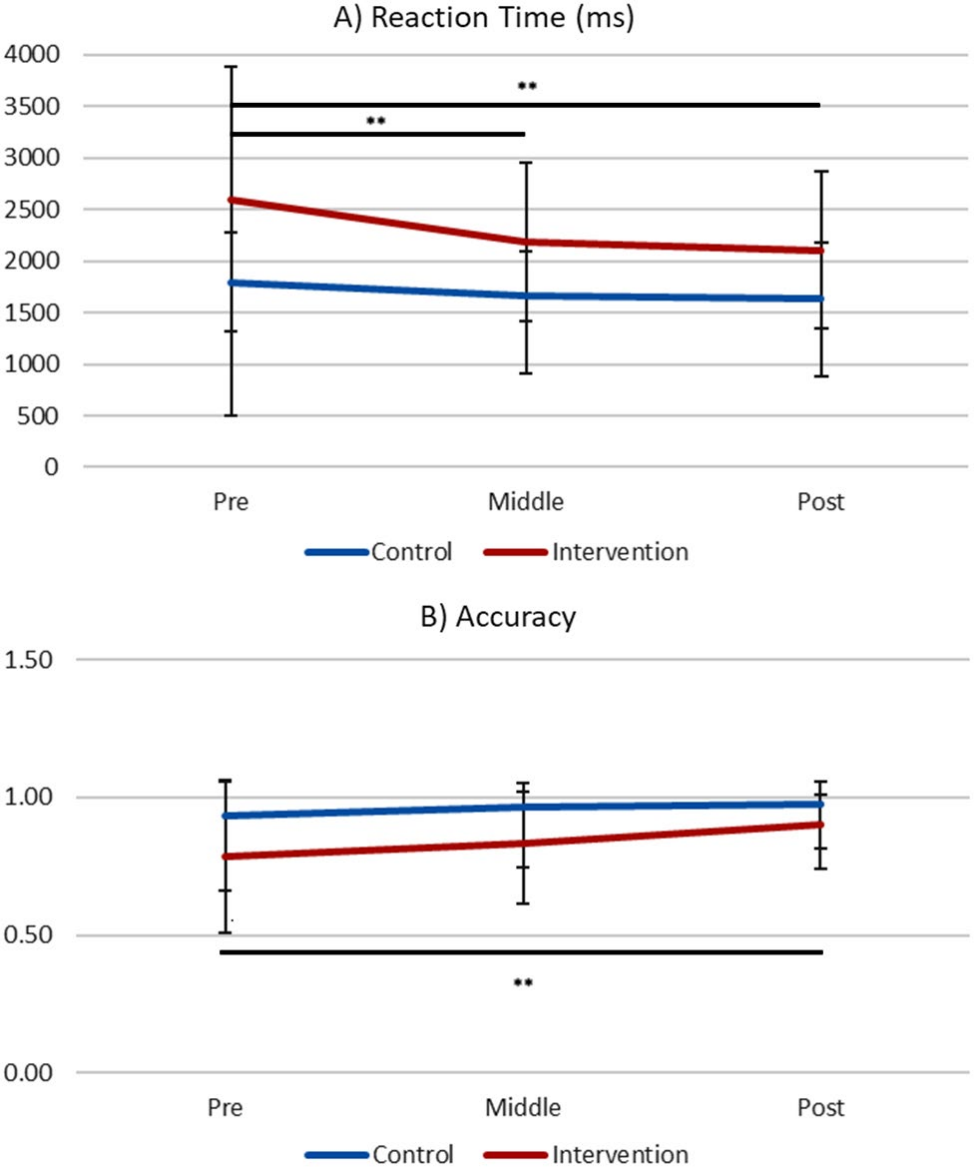
Reaction Time (RT) and Accuracy of Correct Stroop Performance in the Incongruent Condition. The intervention group showed significant improvement in RT (*p* =.002) and accuracy (*p* =.013) from pre to post, where no difference was found for the control group (RT: *p* =.112; accuracy: *p* =.063). *Note*. ms = milliseconds. Error bar: SD. ** p <.01

Only correct trials were included in the reaction time (RT) analyses as it was difficult to interpret RT of incorrect trials. Descriptive statistics of RT (in milliseconds) and accuracy of Stroop performance in the incongruent condition are presented in Table 2; Fig. 1. The main effect of data collection time points on RT was significant in the intervention group (*F*[2,144] = 9.07, *p* <.001, η_p_^2^ = 0.14), but not in the control group (*F*[2,68] = 2.26, *p* =.112, η_p_^2^ = 0.06). Specifically for the intervention group, the RT became faster from baseline to middle (pre: *M* = 2598.239, *SD* = 1281.522; middle: *M* = 2183.263, *SD* = 765.458; *p* =.011, *d* = 0.393), as well as from the baseline to the post (post: *M* = 2105.745, *SD* = 754.787; *p* =.002, *d* = 0.468). The main effect of data collection time points on accuracy was also significant in the intervention group (*F*[2,144] = 5.37, *p* =.006, η_p_^2^ = 0.09), but not in the control group (*F*[2,68] = 2.88, *p* =.063, η_p_^2^ = 0.08). Post hoc tests revealed that accuracy of correct Stroop performance was significantly improved from baseline to post in the intervention group (pre: *M* = 0.785, *SD* = 0.275; post: *M* = 0.899, *SD* = 0.161; *p* =.013, *d* = 0.506).

**Table 2:** Descriptive Statistics of Measured Variables

		Intervention (*n* = 58)				Control (*n* = 35)		
		Pre	Middle	Post		Pre	Middle	Post
Stroop performance in the incongruent condition								
Reaction Time [ms]		2598.24 (1281.52)	2183.26 (765.46)	2105.75 (745.79)		1782.30 (493.08)	1667.69 (428.67)	1629.51 (545.08)
Trial Marking Test [ms]								
TMT-A		76362.53 (28295.07)	68693.45 (28598.82)	64680.26 (23530.48)		57689.63 (28895.47)	54570.06 (15331.08)	53734.91 (20716.87)
TMT-B		132937.16 (64970.10)	112067.55 (64942.19)	105212.84 (63664.67)		88361.24 (37569.05)	75232.74 (29214.66)	74893.97 (37414.43)
TMT-B–TMT-A		61284.09 (56118.49)	49995.70 (55169.02)	43804.98 (51192.29)		30281.00 (32981.81)	20870.65 (22075.08)	21095.79 (23229.79)
Gait Speeds [m/sec.]								
Normal Speed		1.14 (0.20)	1.18 (0.21)	1.22 (0.17)		1.27 (0.18)	1.28 (0.19)	1.33 (0.16)
Maximum Speed		1.41 (0.20)	1.45 (0.23)	1.48 (0.20)		1.68 (0.24)	1.70 (0.25)	1.70 (0.25)
Subjective Vitality [range: 1–7]		5.56 (0.92)	5.88 (0.92)	5.83 (0.98)		5.61 (0.78)	5.70 (0.94)	5.64 (0.89)
Group Cohesion [range: 1–9]								
ATG-T		7.40 (1.37)	7.96 (1.05)	8.14 (0.89)		–	–	–
ATG-S		7.08 (1.30)	7.74 (1.04)	7.74 (1.14)		–	–	–
GI-T		7.01 (1.43)	7.88 (1.16)	8.06 (1.01)		–	–	–
GI-S		6.49 (1.63)	7.37 (1.31)	7.43 (1.35)		–	–	–

*Note*. *Mean* (*SD*). ms = milliseconds; ATG-T = Individual Attractions to the Group-Task; ATG-S: Individual Attractions to the Group-Social; GI-T: Group Integration-Task; GI-S: Group Integration-Social

#### Trial making test

**Fig. 2:**
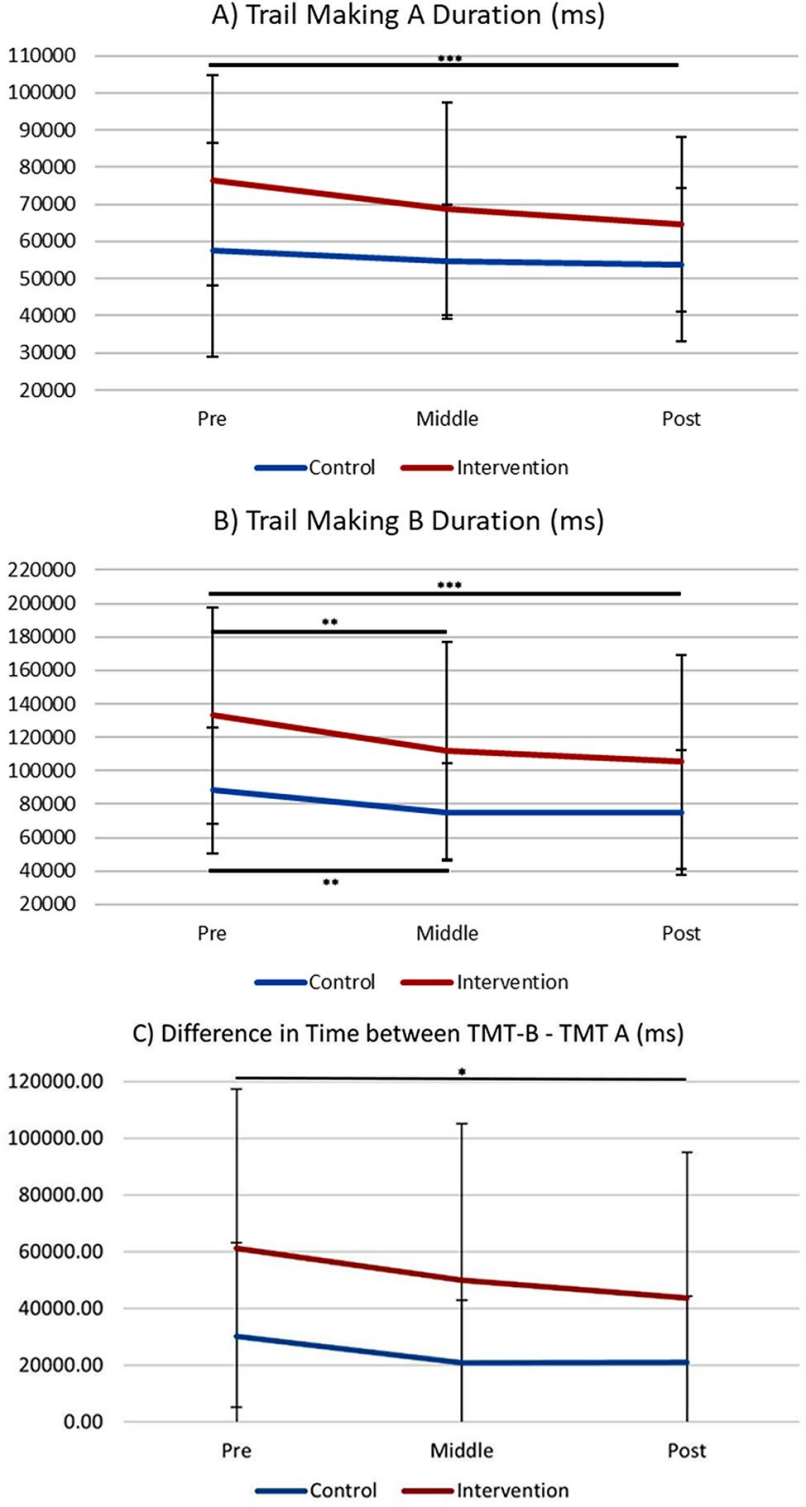
Time (milliseconds) to Complete Trail Making Test A and B. The intervention group showed significant improvement in completing TMT-A (*p* <.001) and TMT-B (*p* =.001) as well as the difference in time between TMT-B and TMT-A (*p* <.05) from pre to post, where no difference was found for the control group (TMT-A: *p* =.369; TMT-B: *p* =.099; TMT-B– TMT-A: *p* =.271). *Note*. ms = milliseconds. Error bar: SD. *** *p* <.001, ** *p* <.0

Descriptive statistics of TMT-A and TMT-B scores are presented in Table 2; Fig. 2. The main effect of data collection time points on the TMT-A duration was significant in the intervention group (*F*[2,144] = 8.41, *p* <.001, η_p_^2^ = 0.13), but not in the control group (*F*[2,68] = 1.01, *p* =.369, η_p_^2^ = 0.06). Post hoc tests indicated that the intervention group took a significantly shorter time to complete the TMT-A from baseline to post (pre: *M* = 76362.53, *SD* = 28295.07; post: *M* = 64680.26, *SD* = 23530.48; *p* <.001, *d* = 0.449). The main effect of data collection time points on the TMT-B duration was significant in the intervention group (*F*[2,86] = 8.93, *p* <.001, η_p_^2^ = 0.17) as well as in the control group (*F*[2,66] = 4.20, *p* =.019, η_p_^2^ = 0.11). Post hoc tests revealed that the intervention group took a significantly shorter time to complete the TMT-B from baseline to middle (pre: *M* = 132937.16, *SD* = 64970.10; middle: *M* = 112067.55, *SD* = 64942.19; *p* <.009, *d* = 0.321) as well as from baseline to post (post: *M* = 105212.84, *SD* = 63664.67; *p* =.001, *d* = 0.431). Although the control group took a significantly shorter duration to complete the TMT-B from baseline to middle (pre: *M* = 88361.24, *SD* = 37569.05; middle: *M* = 75232.74, *SD* = 29214.66, *p* =.011, *d* = 0.390), further improvement was not observed between baseline and post (post: *M* = 74893.97, *SD* = 37414.43, *p* =.099). The main effect of data collection time points on the difference in performance between TMT-B and TMT-A (B – A) was significant in the intervention group (*F*[2, 86] = 3.62, *p* =.031, η_p_^2^ = 0.08), but not in the control group (*F*[2,66] = 1.59, *p* =.212, η_p_^2^ = 0.05). Post hoc tests indicated that TMT-B minus TMT-A (B– A) got significantly smaller from baseline to post (pre: *M* = 61284.09, *SD* = 56118.49; post: *M* = 43804.98, *SD* = 51192.29; *p* <.05, *d* = 0.325).

### Gait speeds

**Fig. 3:**
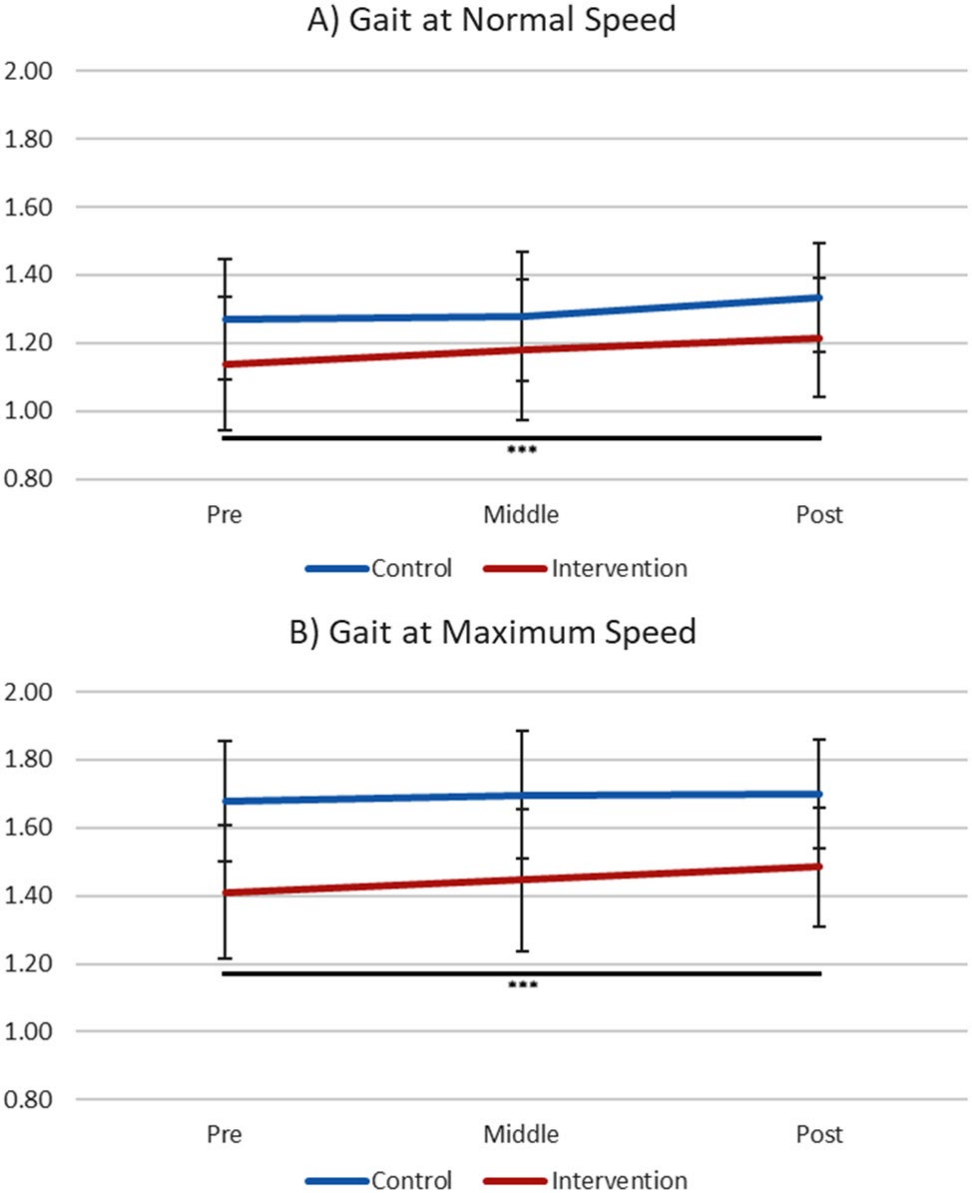
Gait at Normal Speed and Maximum Speed. The intervention group showed significant improvement in gait speed (normal, *p* <.001; maximum, *p* <.001) from pre to post. *Note*. Error bar: SD. *** *p* <.001

Descriptive statistics of gait speeds are presented in Table 2; Fig. 3. The main effect of data collection time points on gait speed at usual pace and at maximum pace was significant in the intervention group (usual pace: *F*[2,114] = 7.51, *p* <.001, η_p_^2^ = 0.12; maximum pace: *F*[2,114] = 9.71, *p* <.011, η_p_^2^ = 0.15), but not significant in the control group (usual: *F*[2,68] = 3.30, *p* =.08, η_p_^2^ = 0.09; maximum: *F*[2,68] = 0.70, *p* =.50, η_p_^2^ = 0.02). Post hoc tests indicated that intervention group’s gait speed at usual pace (pre: *M* = 1.14, *SD* = 0.20; post: *M* = 1.22, *SD* = 0.17; *p* <.001, *d* = 0.431) as well as at maximum pace (pre: *M* = 1.46, *SD* = 0.21; post: *M* = 1.53, *SD* = 0.20; *p* <.001, *d* = 0.341) were significantly improved from baseline to post.

### Subjective vitality

**Fig. 4:**
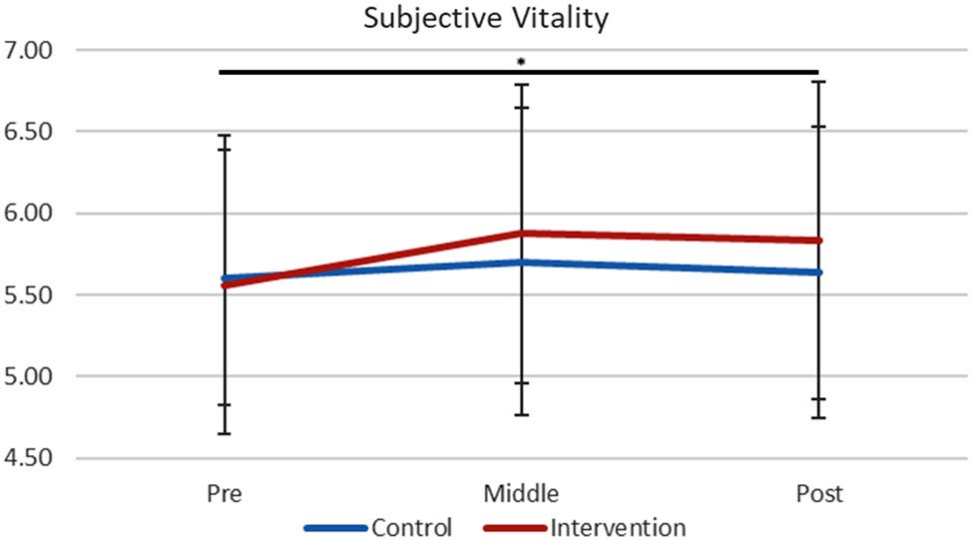
Subjective Vitality Scores. The intervention group showed significant improvement from baseline to post (*p* =.019), but not the control group (*p* =.743). *Note*. Error bar: *SD*. * *p* <.05

The SVS scores in the intervention and control groups are presented in Table 2; Fig. 4. The main effect of data collection time points on the SVS scores was significant in the intervention group (*F*[2,114] = 4.73, *p* =.011, η_p_^2^ = 0.77), but not significant in the control group (*F*[2,68] = 2.99, *p* =.743, η_p_^2^ = 0.01). Post-hoc tests revealed that intervention group’s vitality score significantly increased from baseline to post (pre: *M* = 5.56, *SD* = 0.92; post: *M* = 5.83, *SD* = 0.98; *p* =.019, *d* = 0.284).

### Group Cohesion

**Fig. 5:**
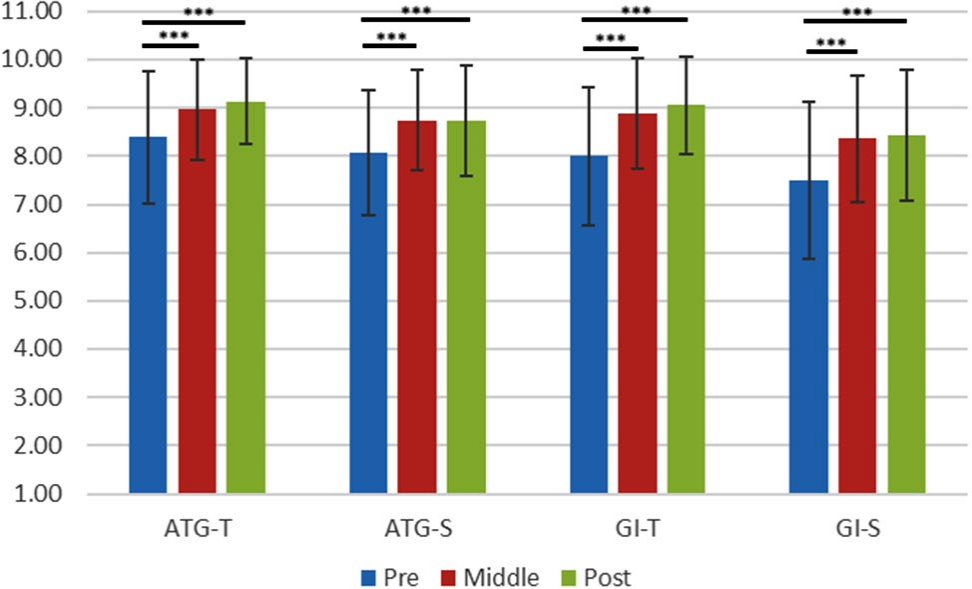
The Averaged Subscale Scores of the Physical Activity Group Environment Questionnaire (PAGEQ) in the Intervention Group show significant improvements across time points (*p* <.001). *Note*. ATG-T: Individual attraction to task factors of group; ATG-S: Individual attraction to social factors of group; GI-T: Group integration based on task; GI-S: Group integration based on social. Error bar: *SD*. *** *p* <.001

The averaged PAGEQ subscale scores in the intervention group are shown in Table 2; Fig. 5. A repeated MANOVA on the PAGEQ subscale scores indicated that the main effect of data collection time points on all the four subscale scores were significant (ATG-T: *F*[2,114] = 18.47, *p* <.001, η_p_^2^ = 0.30; ATG-S: *F*[2,114] = 16.06, *p* <.001, η_p_^2^ = 0.22; GI-T: *F*[2,114] = 12.71, *p* <.001, η_p_^2^ = 0.18; GI-S: *F*[2,114] = 11.55, *p* <.001, η_p_^2^ = 0.18). Post-hoc tests revealed that all the scores significantly increased from baseline to the middle as well as to the post in the intervention group (see Fig. 5).

## Discussion

To ensure the safety and well-being of older adults, it is crucial to have alternative options of conducting SSE in different modes (e.g., online, hybrid, and in-person modes) for older adults and examine the efficacy of the approaches. To this end, the present study investigated the effects of a hybrid SSE on cognitive, physical, psychological, and group functioning in sedentary older adults.

Outcomes of cognitive tests (the Stroop Color-Word Test and the TMT) revealed that the hybrid SSE was highly effective to improve executive function that included working memory, inhibition, and cognitive flexibility [28]. The intervention group showed significant improvement in reaction time and accuracy of correct Stroop performance in the incongruent condition, as well as the TMT-B minus TMT-A (B– A) in the present study. This finding is consistent with several previous studies on in-person SSE and pilot online SSE [10–12, 15–16]. These results suggest that the cognitive challenges included in SSE were properly implemented in the hybrid SSE. In the SSE program, participants need to pay close attention to the instructor’s demonstrations to remember stepping patterns and execute those steps subsequently. They also need to inhibit and modify their natural walking behaviors to finish the demonstrated complex steps accurately [11, 15]. The inhibitory control and fast information processing speed incorporated in the hybrid SSE are useful to prevent falling in daily life settings [8, 11].

Results of physical tests indicated that the hybrid SSE was also beneficial to improve physical functions, such as gait speed at usual pace and at the maximum pace. These results replicated findings of previous in-person SSE studies [5, 6]. Furthermore, the hybrid SSE was effective to enhance subjective vitality, which confirmed the result from a previous pilot study on the online SSE [16].

It is of note that the perception of cohesion by the intervention group in the hybrid SSE were significantly enhanced over the 12 weeks. In the present study, all four PAGEQ subscale scores significantly increased over the intervention period. This means that participants in the intervention group perceived increased engagement and communication with group members in the hybrid SSE task. They also indicated increased feelings of bonding and closeness within the group over the 12 weeks. These results are consistent with some previous studies on in-person SSE [11–12] as well as online SSE [15–16]. The consistent findings confirmed that it is possible to promote different aspects of group cohesion in the hybrid SSE sessions.

## Conclusions

In the present study, SSE was conducted in a hybrid mode amid the COVID-19 pandemic and found to be effective to improve cognitive, physical, psychological, and group functioning in sedentary older adults. These results, which are consistent with previous studies on in-person SSE and pilot online SSE [10–12, 15–16], confirmed that the hybrid SSE is helpful for older adults to gain multiple benefits through the activity. The novel hybrid method and results are the strengths of the present study. Despite the strong points, there are also limitations to the study. For instance, a passive control group was employed in this study. Our original plan was to use an active control group who take part in brisk walking. However, it was impossible to implement the plan due to the challenging circumstances caused by the COVID-19 pandemic. As a passive control group was recruited separately from the intervention group, significant differences in demographics (e.g., age and education) were observed between intervention and control groups. We were thus not able to compare the effects of the hybrid SSE between the groups directly. This is another limitation of the current study and future randomized control trials are encouraged.

Given the coronavirus pandemic circumstance, it was valuable to explore the hybrid SSE approach for older adults. In future research, the multiple beneficial effects of the hybrid SSE for older adults or other populations (e.g., patients [29]) can be further examined by employing an active control group.

## Data Availability

The datasets used and/or analyzed during the current study are available from the corresponding author on reasonable request.
